# The development and characterization of synthetic minimal yeast promoters

**DOI:** 10.1038/ncomms8810

**Published:** 2015-07-17

**Authors:** Heidi Redden, Hal S. Alper

**Affiliations:** 1 Department of Molecular Biosciences, The University of Texas at Austin, 2500 Speedway Avenue, Austin, Texas 78712, USA.; 2 Department of Chemical Engineering, The University of Texas at Austin, 200 E Dean Keeton St. Stop C0400, Austin, Texas 78712, USA.

## Abstract

Synthetic promoters, especially minimally sized, are critical for advancing fungal synthetic biology. Fungal promoters often span hundreds of base pairs, nearly ten times the amount of bacterial counterparts. This size limits large-scale synthetic biology efforts in yeasts. Here we address this shortcoming by establishing a methodical workflow necessary to identify robust minimal core elements that can be linked with minimal upstream activating sequences to develop short, yet strong yeast promoters. Through a series of library-based synthesis, analysis and robustness tests, we create a set of non-homologous, purely synthetic, minimal promoters for yeast. These promoters are comprised of short core elements that are generic and interoperable and 10 bp UAS elements that impart strong, constitutive function. Through this methodology, we are able to generate the shortest fungal promoters to date, which can achieve high levels of both inducible and constitutive expression with up to an 80% reduction in size.

## Abstract (web summary)

Endogenous fungal gene promoters can be hundreds of base pairs long, limiting their use in synthetic biology and biotechnology. Here Redden and Alper screen a library of synthetic promoter elements to generate compact DNA sequences of ∼100 base pairs able to drive high levels of gene expression.

## Graphical abstract

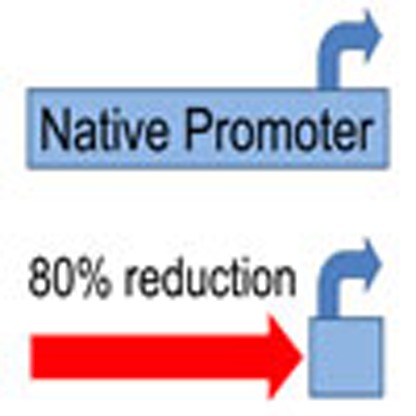

Promoters serve a critical role in establishing baseline transcriptional capacity for nearly every natural and synthetic circuit or pathway^1^,^2^. These elements can synthetically impart controlled, tuneable, inducible, responsive and/or coordinated function of a circuit^3^,^4^,^5^. Such precise control is critical for varied applications from balancing components within a responsive circuit to preventing build-up of toxic intermediate metabolites along a pathway^6^. Thus, promoter elements are indispensable synthetic biology parts and, not surprisingly, are among the first to be annotated and developed for new hosts. However, the field is still limited in the ability to develop a large array of short, purely synthetic promoter constructs that do not rely on native scaffolds. This is particularly important for eukaryotic hosts, such as the yeast *Saccharomyces cerevisiae*. Specifically, since these hosts do not use polycistronic messages, a separate promoter is required for the expression of every gene. Thus, it is desirable to develop promoters with diverse sequences, preferably minimal in length, which are entirely synthetic in nature to reduce endogenous cellular interactions and avoid homologous recombination. Generic workflows for defining and generating such elements are still lacking since most part development approaches are *ad hoc* and rely on native scaffolds.

The innate complexity of eukaryotic transcription makes organisms like *S. cerevisiae* quite distinct from their bacterial counterparts. This complexity is instantiated by considerably longer eukaryotic promoters compared to bacterial ones. *E. coli* promoters typically span well under 100 nucleotides^7^, whereas native yeast promoters (especially those used in synthetic biology efforts) can stretch hundreds of base pairs^8^,^9^. Mechanistically, a longer stretch of DNA is needed to load and stabilize a bulkier (∼100 kDa larger) and more highly regulated eukaryotic RNA polymerase II (RNAP)^10^,^11^. As such, two of the most commonly used yeast promoter elements, the *GAL1* inducible promoter and the strong *GPD* (*TDH3*) promoter span over 400 and 600 nucleotides respectively. Beyond the lack of sequence diversity, these fungal and eukaryotic promoters make large-scale synthetic biology efforts cumbersome. Specifically, a single-gene circuit carrying a 1.5 kb gene requires an additional 1 kb of regulatory DNA (between the promoter and terminator) for appropriate expression, thus increasing the DNA cargo load by over 60%. If an extensive, multi-gene heterologous pathway is required, this regulatory DNA could easily add up to tens of thousands of nucleotides (nearly 10 times as much as needed in bacteria). Thus, minimal-sized fungal promoters are an essential and lacking tool in the field for fungal synthetic biology.

To date, this problem of bulky yeast promoters has been scarcely addressed in synthetic biology; yeast promoter engineering has mainly focused on building from larger, native scaffolds^12^,^13^,^14^,^15^,^16^,^17^,^18^,^19^,^20^,^21^. At the same time, synthetic promoter assembly via hybrid technologies^4^,^22^,^23^ demonstrates a promise in moving away from native scaffolds, but broadly still relies on endogenous parts. Thus, in this study, we address the lack of minimal yeast promoters by creating the shortest yeast promoters to date to expand the potential of synthetic biology in fungi. To develop these minimal promoters, we establish a methodical workflow necessary to identify robust minimal core elements that can be linked with minimal upstream activating sequences (UAS; Fig. 1a). In doing so, we establish promoters that can perform as well as commonly used promoters with up to an 80% reduction in regulatory DNA. Specifically, through a series of rigorous tests, nine robust, minimal core elements with truly modular and context-independent function are identified from a pool of 15 million candidates. These elements are highly unique both among each other and to any native genomic sequences in *S. cerevisiae.* Through an additional library-based screening technique, we demonstrate the facile discovery of minimal (10 bp) UAS elements that can be assembled in a hybrid manner with these minimal core elements to establish short promoters (<120 bp) that can be as strong as the ubiquitously used *GPD* (*TDH3*) promoter as well as other commonly used constitutive promoters in just 1/6th of the DNA length. We likewise demonstrate the ability to establish minimal, inducible promoters through this method with maximal expression levels similar to that of *GAL1*. Thus, this methodology enables the minimization of fungal promoters.

## Results

### Creating a method for identifying minimal yeast promoters

**Figure 1:**
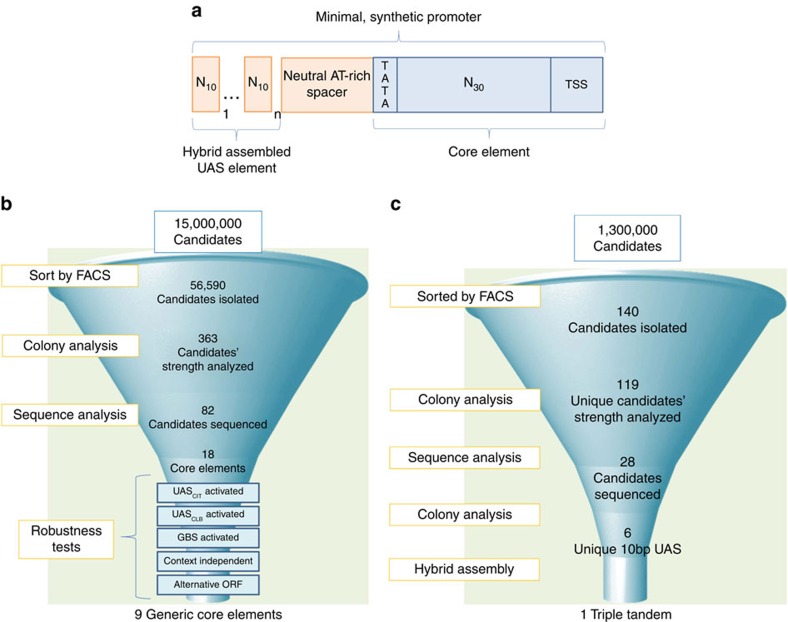
Overview of methodology for developing minimal fungal promoters. (**a**) A schematic of the promoter architecture wherein minimal synthetic promoters were assembled from a library-derived core element (blue), a neutral AT-rich spacer and a hybrid assembly of library-derived UAS elements (red). Element lengths in illustration are to scale. (**b**) A generic workflow for isolating minimal, core promoter elements was followed. Twenty-seven libraries totalling 15 million candidates were created to identify functional core elements. Most promising libraries (0.15%) were isolated by FACS. These sorted cells were subjected to colony analysis via flow cytometry and high-strength candidates were sequenced. Only 18 putative core elements were selected and characterized using a series of robustness tests to arrive at a final set of nine generic, functional core elements. (**c**) One library of 1.3 million 10-bp UAS candidates was analysed and top performers were isolated by FACS. One hundred and nineteen putative candidates were narrowed to just a pool of six UAS after colony analysis, sequencing and robustness tests. Select UAS elements were linked together to demonstrate one highly functioning triple tandem UAS that can establish strong, minimal yeast promoters when linked with a minimal core element.

To design minimal promoters, we established a plasmid-based non-native, core element scaffold (Fig. 1a) to determine the shortest length required for transcription and to serve as a platform for hybrid promoter technology. This core element scaffold was built on distinct, essential sequences for promoter function—a TATA box with consensus sequence of TATAWAWR^24^ followed by a transcription start site (TSS) with consensus sequence of A(A_rich_)_5_NYAWNN(A_rich_)_6_ (ref. 25). The TSS is found in native promoters at a distance of 40–120 bp downstream of the TATA box^25^,^26^. However, to establish minimal promoters, we created and evaluated ensembles of variably spaced TATA box-TSS core elements. Although the core elements contain all sequence components necessary for transcription initiation, very low expression is expected without UAS elements, which provide the overall strength and regulation of a promoter^5^,^27^. Thus, the second module for minimization in our generic scheme (Fig. 1a) is the UAS element positioned upstream of the core region. Traditionally, this element contains transcription factor-binding sites (TFBS) thought to aid in RNAP stabilization and enhanced transcription rates^28^. To minimize these elements, we created and evaluated hybrid assemblies of UAS elements capable of modularly enhancing the expression driven by minimal core elements (Fig. 1a). This methodology led to minimal, yet strong and sequence distinct promoters as described herein. Throughout this workflow, a series of stringency and robustness tests were invoked to establish core elements that are modular, context independent, robust and interoperable (Fig. 1b).

### Core element designs

Our promoter minimization approach first sought to determine a minimal number of nucleotides required between the TATA box and the TSS to promote successful loading of the pre-initiation complex and thus, transcription initiation by RNAP. As previously discussed, native spacing in *S. cerevisiae* is seen to span between 40 and 120 bp (refs 25, 26). This lower limit is peculiar since the structure for yeast RNAP supports a minimal spacing of 30–31 bp (ref. 29) matching the optimal spacing found in mammalian promoters^30^. Thus, multiple plasmid-based spacer libraries were synthesized containing 20 (N_20_), 25 (N_25_) and 30 (N_30_) nucleotides between the TATA box and TSS using random oligonucleotides (Supplementary Fig. 1a,b). The impact of these spacers was assessed by linking these libraries to a fluorescent reporter protein (yECitrine), and assessing library function with flow cytometry. Interestingly, all libraries showed a lengthening in the histogram tails towards higher fluorescence when compared with a negative control (no fluorescent protein; Supplementary Fig. 2a–c). However, only the N_30_ library exhibited a small population shift towards higher fluorescence indicative of a subpopulation of functional core elements (Supplementary Fig. 1b).

As the expected transcription function of core elements is slight, we sought to amplify the signal of functional minimal core elements. First, we established UAS-core element hybrid libraries in an effort to isolate core elements that can be substantially amplified with a UAS (Supplementary Fig. 1a). To do so, we employed a native UAS element previously demonstrated to be effective in yeast, a 275-bp sequence from the mitochondrial citrate synthase gene^22^,^31^ referred to as UAS_*CIT*_. Second, we sought to amplify the signal further by using an expression enhancing terminator, *SPG5*, shown to elevate mRNA concentration by increasing the transcript half-life^32^ (Supplementary Fig. 1c). Both UAS_*CIT*_ and the *SPG5* terminator resulted in shifts in all libraries (Supplementary Fig. 1a,c), with the most dramatic, positive candidate shifts seen in the N_30_ library (Supplementary Fig. 2a–c). As a result, minimal core elements were identified from libraries comprised of UAS_*CIT*_ and *SPG5* terminator linked with the core element. To do so, the top ∼0.15% expressing cells of every library was sorted by fluorescence-activated cell sorting (FACS) and subsequently only the N_30_ library resulted in robust promoters with a low frequency of multiple insertions (Supplementary Fig. 2d–f).

### Isolation of putative cores elements

From the initial 15 million candidates synthesized as described above, we sought to isolate minimal core elements with desirable characteristics for synthetic biology applications. Specifically, these core elements should (1) be generically activated by any UAS or TFBS, (2) function with alternative genes and (3) display little context dependence. Thus, a series of robustness tests were used to narrow down the candidates to a set of nine robust generic core elements (Fig. 1b).

**Figure 2:**
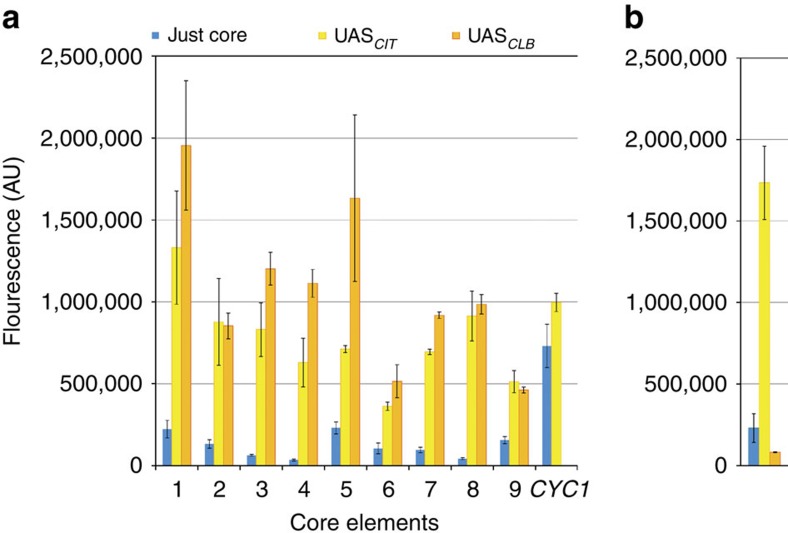
UAS activation of synthetic core elements creates constitutive promoters. (**a**) Core elements were tested with two known constitutive UAS elements, UAS_*CIT*_ and UAS_*CLB*_. All of the final nine minimal core elements were sufficiently activated by each of these elements (*n*=4). Quadruplicates used in an effort to reduce error. (**b**) For demonstrative purposes, one core element rejected in this robustness test is shown (*n*=3). Error bars represent s.d. among biological replicates as indicated.

To begin, the candidates were isolated from the enriched UAS_*CIT*_-N_30_-*SPG5* library obtained via FACS followed by isolated colony analysis, and sequencing. As noted earlier, this library had the lowest frequency of multiple insertions (Supplementary Fig. 2f) and thus, indicates that 30 bp may be the minimal spacing required between the TATA box and TSS for *S. cerevisiae*. Following isolation, characterization, quality control (reproducible on retransformation and homogenous histograms) and sequencings, a total of 18 putative unique core elements were identified. As these elements were currently linked with a UAS_*CIT*_ upstream region, we removed this region and assessed the strength of the core element itself and found that indeed these core elements allowed for slight, but detectable, transcription (Fig. 2a).

### Isolation of robust and minimal core promoter elements

These 18 putative core elements were next assessed through a series of robustness tests (Fig. 1b). All fluorescence values compared with one another in this manuscript were gathered on the same day to mitigate day-to-day absolute fluorescence variations. To begin, we sought to evaluate the impact of an alternative, constitutive UAS. To do so, these core elements were linked with another previously used UAS^22^, the 240-bp sequence of the mitotic cyclin (*CLB2*) gene^33^, termed the UAS_*CLB*_. Many of the putative core elements were also able to be activated by UAS_*CLB*_ with a success threshold of at least twofold increase in fluorescence (Fig. 2a); however, some were not activated by this UAS and thus were removed from the candidate pool (Fig. 2b). Of the 18 putative core elements, three were determined to not be functionally robust with respect to activation by an alternative, constitutive UAS element.

**Figure 3:**
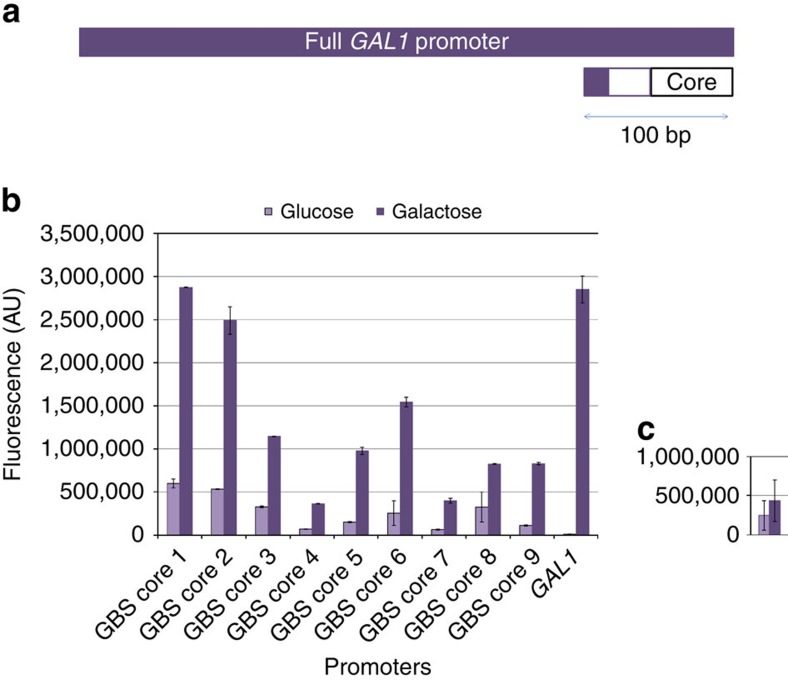
TFBS activation of synthetic core elements creates inducible promoters. (**a**) Core elements were paired with a minimal GBS. Specifically shown here is UAS_*G4BS4*_ to develop short, minimal galactose-inducible promoters. Lengths of promoters are illustrated to scale. Scale bar, 100 bp. (**b**) When linked with certain core elements, the GBS was able to establish short, galactose-inducible promoters with a fully induced strength comparable to that of the *GAL1* native promoter. (**c**) Of the 18 core elements tested in this construct, two did not activate with both UAS_*G4BS3*_ and UAS_*G4BS4*_. For demonstrative purposes, one rejected core element is shown here, which does not activate with UAS_*G4BS4*_. Error bars represent s.d. among biological triplicate.

As a second robustness test for generalizability of these cores, we sought to demonstrate whether these core elements could be linked with a minimal galactose-inducible UAS element to enable inducible promoter function in a minimal sequence space (Fig. 1b). To do so, these 18 candidate core elements were linked with 17 bp *GAL1*-derived Gal4p-binding sites (GBS), UAS_*G4BS3*_ and UAS_*G4BS4*_, as previously described^22^. As a means of linking these minimal core elements to a short UAS element, we evaluated variable spacing between the TATA box of the core element and the TFBS, and found that a neutral AT-rich 30 bp sequence (Supplementary Fig. 3a and Supplementary Fig. 4a,b) was required to avoid possible observed steric hindrances between the Gal4p TF and TATA-box-binding protein component of pre-initiation complex (Supplementary Fig. 3b,c). In general, we found UAS_*G4BS4*_ induced expression under galactose better than UAS_*G4BS3*_ (Supplementary Fig. 5) with both UAS producing little to no effect under glucose (Supplementary Fig. 6). As with the UAS_*CLB*_, most (Fig. 3a,b), but not all (Fig. 3c), putative core elements were able to be turned into inducible promoters with the addition of a UAS_*G4BS4*_. Of the 18 putative core elements, two were determined to not be functionally robust with respect to activation by both inducible UAS elements tested. More importantly, when UAS_G4BS4_ is combined with minimal core elements, the expression level of the fully induced synthetic promoters is comparable to that of the full native *GAL1* promoter in two of the cases, but in only 22% of the DNA sequence (Fig. 3a,b). Furthering on this, when a 54-bp broader UAS element (found in native Gal1 and containing three Gal4p-binding sites) is linked with all nine of these final core elements, most either reach the fully induced strength of the full-length *GAL1* promoter (Supplementary Fig. 7). Although all the inducible promoters constructed still have some glucose leakiness when compared with that of full Gal1 promoter (presumably due to the lack of a glucose repression sequence), the ability to even construct these promoters, especially with high expression capacity in induced mode, highlights the potential of minimal promoter construction.

As a third robustness test, we sought to determine context dependency of core elements linked with UAS_*CIT*_ and UAS_*G4BS4*_, as well as without any UAS (Fig. 1a). Promoters with limited context dependency provide a predictable and orthologous tool for simplified synthetic biology. Thus, to ensure our set of core elements is context independent, we applied a simple test; we analysed their expression strengths by flipping the entire expression cassette and reintroducing it back into the same plasmid location (Supplementary Fig. 8a). Although this test does not confirm or guarantee complete orthogonality, it certainly provides some evidence for a more context independent promoter, especially when compared with some commonly used, endogenous elements. Prior evidence within our lab supports the finding that native promoters (including *CYC1* shown in Supplementary Fig. 8a) can demonstrate drastically different transcriptional profiles in light of this test, thus highlighting the importance of developing context independent parts. Of the 18 putative core elements, five were determined to not function in a context-independent manner making this test the most stringent one applied to the core elements (Supplementary Fig. 8b).

As a final robustness test, we sought to evaluate the impact of these core elements with different genes. Thus, an alternative reporter gene was used to ensure that promoters could be successfully applied to other expression systems. Using *LacZ* as an alternative ORF, we demonstrate that all core elements with and without a UAS_*CIT*_ can induce the transcription of *LacZ* in a similar fashion as the fluorescent protein (Supplementary Fig. 9). Furthermore, we also show that fluorescent levels measured with flow cytometry correlate well with mRNA abundance levels indicating that the function of these elements is indeed at the transcription level (Supplementary Fig. 10). Thus, core elements isolated via this scheme were seen to function independently of the downstream gene.

### Final set of nine minimized generic core elements

**Figure 4:**
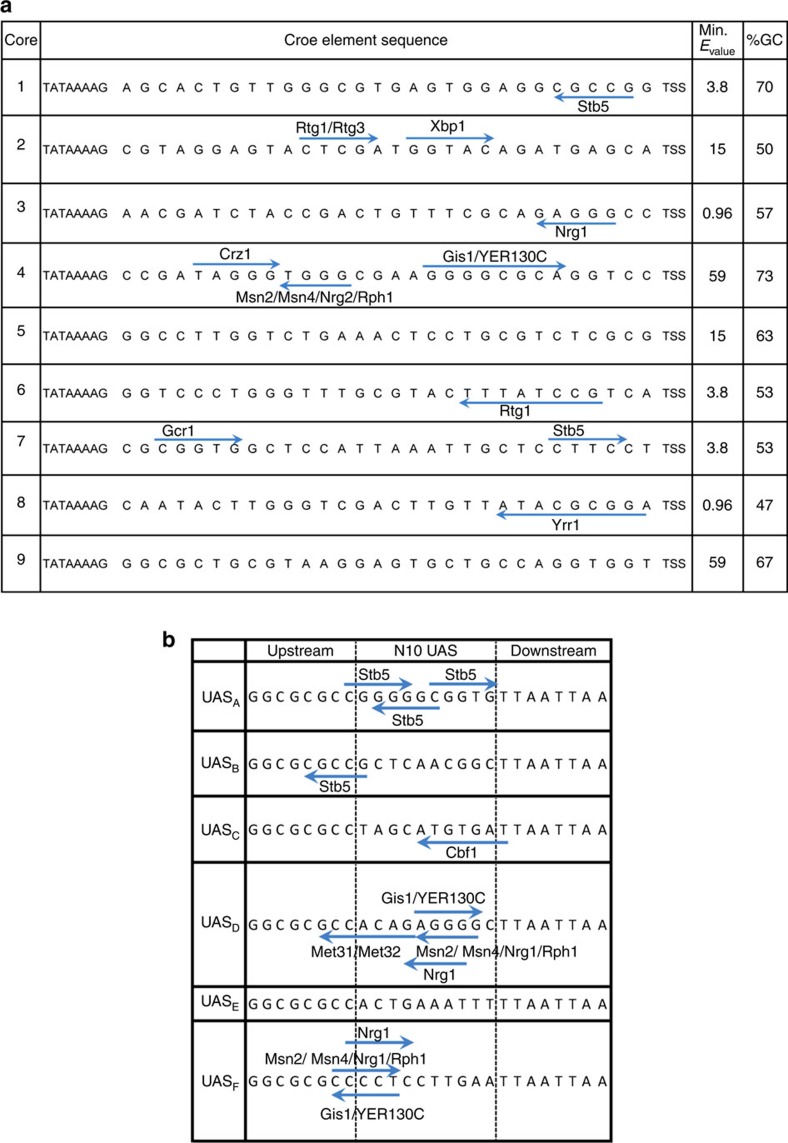
Minimal core elements and UAS sequences. TFBS are indicated by arrows with direction of arrow designating the orientation of site. Protein predicted to bind to these sites are labelled as so. (**a**) The final set of nine core elements established here are distinct from one another spanning a %GC content of 47–73. The quantity, quality and directionality of predicted TFBS, as determined by YEASTRACT, vary greatly. These promoters also display varying dependences to SAGA complex. In a Δ*spt3* strain, strengths of core elements assembled in three promoter contexts (just core, with UAS_*CIT*_ and with UAS_*G4BS4*_) were determined. SAGA dependency score of 3 indicates that all three promoters' strengths were affected in this knockout strain, while a score of 0 indicates that none of the promoters' strengths were disrupted. High minimum *E*_value_ obtained from BLAST confirm core elements' uniqueness. (**b**) Six 10 bp minimal UAS sequences isolated through the library-based sorting and selection.

In the workflow described above, we sorted through 15 million elements to isolate 18 putative core elements. Through a series of robustness tests to isolate core elements with desirable characteristics that could be used in synthetic biology efforts, we narrowed our useable set to just nine core elements (Fig. 1b). This indicates that not only do most random sequences between TATA box and TSS fail to function properly, but that many core elements are unable to function in a generic manner (especially with respect to genetic context), highlighting the exceptionality of the core elements selected in this research. Although the core elements share common desirable characteristics at the phenotypic level, their sequences are quite dissimilar spanning a wide range of GC content from 47 to 70% (Fig. 4a), and possessing a diversity of TFBS, both in quantity, quality and directionality as assessed by YEASTRACT^34^ (Fig. 4a). This high GC content makes these promoters quite distinct from endogenous sequences (especially of strong promoters like *TDH3*). Finally, the sequence homology to the genome is low among the set as none of them match any sequences found in the genome of *S. cerevisiae* (Fig. 4a). Thus, this set of nine minimal core promoters are functionally similar and robust, yet sequence diverse.

### Minimal UAS elements can create strong and short promoters

Our original promoter scaffold required both minimal core and UAS elements to obtain a short, highly functional promoter. The results of our galactose-inducible promoter test above demonstrate the potential to get extremely high transcriptional activity (matching the value of *GAL1* promoter, the highest expression level in yeast) in only 22% of the DNA space. However, for the case of constitutive promoters, there are no known short UAS elements. Thus, we turned to a generalizable workflow to identify minimal UAS elements (Fig. 1a,c). The initial, minimal hypothetical length for these UAS elements is the span of a TFBS and as most TFBS are within 10 bp (ref. 35), we hypothesized that it was possible to create minimal, constitutive 10-bp UAS elements.

**Figure 5:**
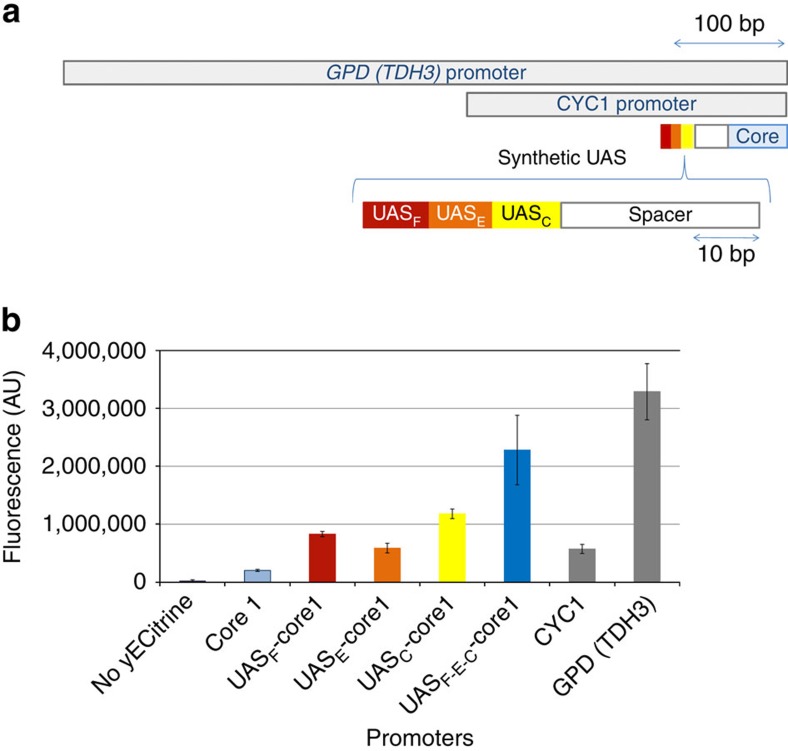
Fully synthetic minimal promoters can drive high expression. By linking minimal UAS elements with a core promoter element, synthetic promoters approaching *GPD* strength can be assembled. (**a**) Lengths of promoters are illustrated to scale. Scale bar is provided. All synthetic UAS shown (UAS_F_, UAS_E_ and UAS_C_) are positioned upstream of the core element using a singular AT-rich neutral 30 bp spacer. Size scale bars are provided. (**b**) All synthetic UASs can activate core element to levels comparable to the strength of the commonly used promoters *CYC1*. When assembled in a hybrid fashion, tandem UAS elements can amplify the strength of these core elements with strengths approaching *GPD* (*TDH3*) in <20% of the DNA. Error bars represent s.d. among the biological triplicate.

In similar fashion to the core elements, a random N_10_ library was synthesized upstream of minimal core 1 (Fig. 1a). To enable sufficient binding with a potential TF, we established the same neutral spacer used to distance GBS from the core (Supplementary Fig. 3a) as described above. In this scheme, a potential TFBS positioned ∼100 bp from the TSS would follow a general architecture of promoters^36^,^37^. Thus, a plasmid-based library of 1.3 million elements was narrowed to 140 candidates using FACS (Fig. 1c). This enriched library was subjected to isolated colony analysis and sequencing (Fig. 1c). We determined that many of the 10-bp UAS isolated in this manner were highly sensitive to growth phase, a characteristic we would expect if the elements were recruiting variably expressed TFs. In fact, YEASTACT was able to identify at least one putative TFBS in 15 out of the 28 UAS sequenced. Overall, of the 119 initially isolated 10-bp UAS elements, only six were consistent and robust in strength (Fig. 1c). Moreover, using this methodology, we were able to isolate 10 bp sequences that could independently activate our minimized core element to expression levels above the commonly used promoter *CYC1* (Fig. 5b). Moreover, these minimal UAS elements can function as well as much longer UAS elements such as UAS_*CIT*_(Supplementary Fig. 11). As a further evaluation of robustness, we demonstrate that these UAS elements are generic enough to activate other core elements, such as core element 2 (Supplementary Fig. 11). Finally, to begin to evaluate these constructs in alternative contexts, we performed a genomic integration of several of the single UAS-based synthetic promoters. We demonstrate that these minimal UAS linked with a core element can function in the genome in a relatively predictable manner (Supplementary Fig. 12).

Finally, we sought to demonstrate how these functional minimal UAS elements can be linked in tandem to establish high-strength, short promoters in yeast. To do so, we hybrid assembled various UAS elements (Fig. 1a) in tandem to find that a combination of three of these elements can rapidly achieve 70% of the strength of the strongest constitutive yeast promoter, *GPD* (*TDH3*), in only 18% of the DNA space (Fig. 1a,b) and exceed the strength of almost every other used constitutive yeast promoter. Thus, through this methodology, it is possible to minimize UAS element from hundreds of base pairs to just ten. By assembling these minimal UAS elements in a hybrid manner to minimal cores elements, we were able to establish strong promoters and reduce the DNA burden of a promoter over 80%.

## Discussion

Before this study, yeast promoters required hundreds of base pairs to achieve high transcriptional capacity. Through the methodical workflow developed here, it was possible to identify robust, minimal core elements that can be linked with minimal 10 bp UAS elements to establish high-strength promoters (with over 80% reduction in size). The resulting promoters approach a size comparable to bacterial host systems with strength comparable to large fungal promoters. Moreover, the framework developed here can allow for continued advancements in minimization of orthologous synthetic promoters in fungal hosts.

The nine minimal core elements described here were identified from a pool of 15 million candidates (Fig. 1b). On a sequence basis, each of these minimal core elements is distinct from both each other and the native *S. cerevisiae* sequence. To further elucidate the potential distinct mechanisms of these elements, it was hypothesized that many of these synthetic promoters created with these core elements would use the SAGA complex. A critical component of the SAGA complex is the Spt3p subunit that is essential for transcriptional activation^38^,^39^. Testing the SAGA complex interaction of core elements hybridized with a UAS (UAS_*CIT*_, UAS_*G4BS4*_ ) and without a UAS was done in a *Δspt3* strain. Whereas the Δ*spt3* mutation removed the functionality of the *GAL1* native promoter as expected^40^, the impact on each of these core elements by themselves, and assembled into assorted promoters varied (Fig. 4a). While this experiment does not conclusively prove linkage to Spt3p-dependent transcription, the marked difference for each promoter suggest that different transcription initiation machinery is utilized across this set of core elements. This characteristic makes this set an excellent tool for generating diverse promoter engineering variants.

We illustrate the capacity of the core elements to function in a promoter by assembling them with library-derived UAS elements and thus creating strong, constitutive minimal promoters. From 1.3 million 10-bp UAS candidates, we isolated a set of six constitutive 10 bp UAS elements. These elements, like the core elements, are also distinct in sequence from each other and were assessed via robustness analysis (Fig. 4c). In addition to being able to generically activate multiple core elements, an analysis based on nucleosome prediction software NuPop^41^ demonstrates features consistent with our prior efforts to generate stronger promoters^20^. Specifically, these six minimal UAS elements induce a predicted narrow, yet sharp nucleosome-depleted region at the TSS (Supplementary Fig. 13). Furthermore, additional hybrid assemblies of 10 bp UAS elements as performed in this work extends the predicted nucleosome depletion region upstream, a pattern consistent with native promoters^42^ (Supplementary Fig. 13). Interestingly, YEASTRACT database search identifies multiple possible TFBS in these UAS, which may operate in concert with possible nucleosome depletion in these promoters to create accessible, functional binding sites and hence more functional promoters^43^,^44^,^45^(Fig. 4b).

Finally, this workflow highlights the importance of robustness tests for promoters. Specifically, we find that generic and interoperable promoters require both robustness to alternative UAS elements and especially genomic context. Through this work here, half of the putative core elements failed these robustness tests and were thus eliminated from the final collection. The most stringent test, context flipping, demonstrates the importance of genomic context in performance (and often, mischaracterization) of synthetic parts^46^. Such robustness analysis is important for future synthetic part evaluation. Moreover, the largest culling seen in the putative pool came at the colony analysis level. Specifically, constructs simply identified through FACS did not always function in a robust manner with respect to growth phase, homogenous expression and retransformation.

With this work, we are able to construct a constitutive promoter with a strength approaching that of the ubiquitous yet lengthy 655 bp *GPD* (*TDH3*) in just 116 bp. When these minimal promoters are combined with minimal terminators^47^, the overall regulatory DNA load of an expression cassette can be reduced by 80–90%—an important feature for improving the ease of larger-scale synthetic systems in yeast. This workflow presented here was able to create minimal, robust, orthologous promoters and expands the potential for yeast promoter engineering. Moreover, this generic workflow can be followed in alternative fungal hosts to further expand the synthetic biology toolbox.

## Methods

### Strains and media

p416 yeast expression vectors were propagated in *Escherichia coli* DH10β. *E. coli* strains were cultivated in LB medium^48^ (Teknova) at 37 °C with 225 r.p.m. orbital shaking. LB was supplemented with 50 μg ml^−1^ ampicillin (Sigma) for plasmid maintenance and propagation. Yeast strains (BY4741 and BY4741 Δspt3 obtained from Euroscarf and Open Biosystems respectively) were cultivated on a yeast synthetic complete medium containing 6.7 g of yeast nitrogen base (Difco) l^−1^, 20 g glucose l^−1^ and a mixture of amino acids, and nucleotides without uracil (CSM, MP Biomedicals, Solon, OH). All medium was supplemented with 1.5% agar for solid media.

For *E. coli* transformations, 50 μl of electrocompetent E. coli DH10β^48^ were mixed with 50 ng of ligated DNA and electroporated (2 mm Electroporation Cuvettes (Bioexpress) with Biorad Genepulser Xcell at 2.5 kV. Transformants were recovered in 1 ml SOC Medium (Cellgro), plated on LB agar and incubated overnight at 37 °C. Single clones were amplified in 2 ml LB medium and incubated overnight at 37 °C. Plasmids were isolated (QIAprep Spin Miniprep Kit, Qiagen) and confirmed by sequencing.

For yeast transformations, 20 μl of chemically competent *S. cerevisiae* BY4741 were transformed with 1 μg of each appropriate purified plasmid using Frozen EZ Yeast Transformation II Kit (Zymo Research, Irvine, CA, USA) according to the manufacturer's instructions. Transformations were plated on CSM-Ura plates, and incubated for 2 days at 30 °C. Single colonies were picked at random into 2 ml of CSM-Ura liquid media and incubated at 30 °C for 2 days. Yeast and bacterial strains were stored at −80 °C in 15% glycerol. Plasmids from yeast were isolated using Zymoprep Yeast Plasmid Miniprep II kit.

### Cloning procedures

All p416 plasmids were assembled using restriction enzyme-based cloning techniques. Oligonucleotides were purchased from Integrated DNA Technologies (Coralville, IA). Sequences and details can be found in Supplementary Table 1. PCR and double-stranding reactions were performed with Phusion DNA Polymerase from New England Biolabs (Ipswich, MA) according to the manufacturer specifications. Digestions were performed according to manufacturer's (NEB) instructions. PCR products and digestions were cleaned with a QIAquick PCR Purification Kit (Qiagen). Phosphatase reactions were performed with Antarctic Phosphatase (NEB) according to the manufacturer's instructions and heat inactivated for 10 min at 65 °C. Ligations (T4 DNA Ligase, Fermentas) were performed for 3–18 h in 5:1–10:1 insert to backbone ratio at 16–22 °C followed by heat inactivation at 65 °C for 10 min.

### Library preparation

Core element libraries and UAS library assembled are listed in Supplementary Table 2. Double-stranded oligonucleotide libraries of N_20_, N_25_ and N_30_ for core element libraries and N_10_ UAS were created using oligonucleotides listed in Supplementary Table 1. Double stranding was performed using Phusion DNA Polymerase from New England Biolabs with a touchdown annealing step followed by a 5-min 72 °C elongation step in thermocycler. All core element libraries and UAS library were cloned into the HindIII/XbaI site and AscI/PacI site respectively in p416 using cloning techniques previously mentioned unless where noted in following text. All libraries were ligated in a 3:1 ligation ratio with 2 μg of backbone in 20-μl reaction volume. Library ligations were desalted for 10 min on nitrocellulose membrane filters (MF 0.025 μm VSWP membrane filters) after 24 h of ligation at 16 °C. Entire ligation mixture was transformed into freshly prepared electrocompetent *E. coli* DH10β^48^ and plated onto LB plates. *E. coli* colonies were counted, scraped, and plasmids were isolated (QIAprep Spin Miniprep Kit, Qiagen) and transformed into freshly prepared BY4741 (ref. 49). *E. coli* colony counts can be found in Supplementary Table 2 as library size. After 48 h of flask growth, aliquots of each library covering five times the size of the yeast library in terms of number of cells were stored at −80 °C in 15% glycerol.

### Flow cytometry and FACS

Yeast cultures were started in triplicate from glycerol stock, and were grown for 2 days to stationary phase. All yeast cultures were inoculated at an OD_600_ of 0.01 and grown to an OD_600_ of 0.7–0.9 in a 30-°C shaker. ΔSpt3 BY4741 (Fischer Scientific) strains under galactose growth were inoculated at OD of 0.10 due to lack of consistent growth at lower OD inoculations. Data collected for Fig. 2 were run in quadruplicate and grown in a 96-deep well block, 1 ml culture volume. Fluorescence was analysed (LSRFortessa Flow Cytometer, BD Biosciences) at an excitation wavelength of 488 nm and detection wavelength of 530 nm. A total of 10,000 events were gathered at a flow rate of 2,000 events s^−1^. An average fluorescence and s.d. were calculated from the mean values for the biological replicates. Flow data were collected on the same day to mitigate possible variations in day-to-day fluorescence values. Flow cytometry data were analysed using FlowJo software. Top ∼0.15% of a million yeast cells from each library were sorted using BD FACS Aria Cell sorter. Cells counted by the FACS instrument are listed in Supplementary Table 2. Sorted cells were grown for 24 h at 30 °C in 2 ml CSM-Ura media at 225 r.p.m. At least 10 times the amount of cells isolated were plated onto CSM-Ura as isolated from the sorting. Colonies were randomly selected from plates and grown for 2 days to stationary phase in 2 ml CSM-Ura. Yeast cultures were inoculated at an OD_600_ of 0.01 and grown to an OD_600_ of 0.7-0.9 in a 30-°C shaker. Fluorescence was analysed (LSRFortessa Flow Cytometer, BD Biosciences) and highly fluorescent cultures were streaked onto plates, picked in triplicate and glycerol stocked. Flow analysis was performed again in triplicate as previously described to ensure robustness.

### qPCR assay

Yeast cultures were grown to OD_600_ of 0.7 to 0.9. Fluorescence was measured simultaneous with RNA extraction of 500 μl of culture (Quick-RNA Miniprep, Zymo Research Corporation). RNA was reverse transcribed (High Capacity cDNA Reverse Transcription Kit, Applied Biosystems) and quantified in triplicate (SYBR Green PCR Master Mix, Life Technologies) after RNA extraction. Transcript levels were measured relative to that of a housekeeping gene (ALG9; Viia 7 Real Time PCR Instrument, Life Technologies). Primers used for quantification are listed in Supplementary Table 1.

### LacZ assay

Yeast cultures were grown from triplicate glycerol stock for 2 days. Cultures were inoculated at 0.01 OD and grown overnight to OD_600_ of 0.7 to 0.9. Cells were mixed with appropriate reagents and incubated according to instructions (AB Gal-Screen System). Chemiluminescent signal was measured with Biotek Cytation 3 imaging reader.

### gDNA integrations

Expression cassettes were cloned into p406. Ura3 marker of p406 was cleaved with BstBI restriction enzyme following manufacturer's instructions. Expression cassettes were integrated into BY4741 genome^50^.

## Additional information

**How to cite this article:** Redden, H. *et al*. The development and characterization of synthetic minimal yeast promoters. *Nat. Commun.* 6:7810 doi: 10.1038/ncomms8810 (2015).

## Supplementary Material

Supplementary Information Supplementary Figures 1-13 and Supplementary Tables 1-2

## References

[b1] Keaveney M. & Struhl K. Activator-mediated recruitment of the RNA polymerase II machinery is the predominant mechanism for transcriptional activation in yeast. Mol. Cell 1, 917 (1998). 9660975 10.1016/s1097-2765(00)80091-x

[b2] Tirosh I., Barkai N. & Verstrepen K. Promoter architecture and the evolvability of gene expression. J. Biol. 8, 95 (2009). 20017897 10.1186/jbiol204 PMC2804285

[b3] Teo W. S. & Chang M. W. Development and characterization of AND-gate dynamic controllers with a modular synthetic GAL1 core promoter in Saccharomyces cerevisiae. Biotechnol. Bioeng. 111, 144 (2014). 23860786 10.1002/bit.25001

[b4] Liang J., Ning J. C. & Zhao H. Coordinated induction of multi-gene pathways in Saccharomyces cerevisiae. Nucleic Acids Res. 41, e54 (2012). 23262224 10.1093/nar/gks1293 PMC3581276

[b5] Afonso B., Silver P. A. & Ajo-Franklin C. M. A synthetic circuit for selectively arresting daughter cells to create aging populations. Nucleic Acids Res. 38, 2727 (2010). 20150416 10.1093/nar/gkq075 PMC2860115

[b6] Lubliner S., Keren L. & Segal E. Sequence features of yeast and human core promoters that are predictive of maximal promoter activity. Nucleic Acids Res. 41, 5569 (2013). 23599004 10.1093/nar/gkt256 PMC3675475

[b7] Smanski M. J. . Functional optimization of gene clusters by combinatorial design and assembly. Nat. Biotechnol. 32, 1241 (2014). 25419741 10.1038/nbt.3063

[b8] Singh V. Recent advancements in synthetic biology: current status and challenges. Gene 535, 1 (2014). 24269673 10.1016/j.gene.2013.11.025

[b9] Redden H., Morse N. & Alper H. S. The synthetic biology toolbox for tuning gene expression in yeast. FEMS Yeast Res. 15, 1 (2014). 10.1111/1567-1364.12188 25047958

[b10] Zhang G. & Darst S. A. Structure of the *Escherichia coli* RNA polymerase α subunit amino-terminal domain. Science 281, 262 (1998). 9657722 10.1126/science.281.5374.262

[b11] Cramer P., Bushnell D. A. & Kornberg R. D. Structural basis of transcription: RNA polymerase II at 2.8 ångstrom resolution. Science 292, 1863 (2001). 11313498 10.1126/science.1059493

[b12] Alper H., Fischer C., Nevoigt E. & Stephanopoulos G. Tuning genetic control through promoter engineering. Proc. Natl Acad. Sci. USA 102, 12678 (2005). 16123130 10.1073/pnas.0504604102 PMC1200280

[b13] Du J., Yuan Y., Si T., Lian J. & Zhao H. Customized optimization of metabolic pathways by combinatorial transcriptional engineering. Nucleic Acids Res. 40, e142 (2012). 22718979 10.1093/nar/gks549 PMC3467037

[b14] Nevoigt E. . Engineering of promoter replacement cassettes for fine-tuning of gene expression in *Saccharomyces cerevisiae*. Appl. Environ. Microbiol. 72, 5266 (2006). 16885275 10.1128/AEM.00530-06 PMC1538763

[b15] Blount B. A., Weenink T., Vasylechko S. & Ellis T. Rational diversification of a promoter providing fine-tuned expression and orthogonal regulation for synthetic biology. PLoS ONE 7, e33279 (2012). 22442681 10.1371/journal.pone.0033279 PMC3307721

[b16] Jeppsson M., Johansson B., Jensen P. R., Hahn-Hägerdal B. & Gorwa-Grauslund M. F. The level of glucose-6-phosphate dehydrogenase activity strongly influences xylose fermentation and inhibitor sensitivity in recombinant *Saccharomyces cerevisiae* strains. Yeast 20, 1263 (2003). 14618564 10.1002/yea.1043

[b17] Ligr M., Siddharthan R., Cross F. R. & Siggia E. D. Gene expression from random libraries of yeast promoters. Genetics 172, 2113 (2006). 16415362 10.1534/genetics.105.052688 PMC1456374

[b18] Iyer V. & Struhl K. Poly(Da-Dt), a ubiquitous promoter element that stimulates transcription via its intrinsic DNA-structure. EMBO J. 14, 2570 (1995). 7781610 10.1002/j.1460-2075.1995.tb07255.x PMC398371

[b19] Raveh-Sadka T. . Manipulating nucleosome disfavoring sequences allows fine-tune regulation of gene expression in yeast. Nat. Genet. 44, 743 (2012). 22634752 10.1038/ng.2305

[b20] Curran K. A. . Design of synthetic yeast promoters via tuning of nucleosome architecture. Nat. Commun. 5, 4002 (2014). 24862902 10.1038/ncomms5002 PMC4064463

[b21] Sharon E. . Inferring gene regulatory logic from high-throughput measurements of thousands of systematically designed promoters. Nat. Biotechnol. 30, 521 (2012). 22609971 10.1038/nbt.2205 PMC3374032

[b22] Blazeck J., Garg R., Reed B. & Alper H. S. Controlling promoter strength and regulation in *Saccharomyces cerevisiae* using synthetic hybrid promoters. Biotechnol. Bioeng. 109, 2884 (2012). 22565375 10.1002/bit.24552

[b23] Khalil Ahmad S. . A synthetic biology framework for programming eukaryotic transcription functions. Cell 150, 647 (2012). 22863014 10.1016/j.cell.2012.05.045 PMC3653585

[b24] Basehoar A. D., Zanton S. J. & Pugh B. F. Identification and distinct regulation of yeast TATA box-containing genes. Cell 116, 699 (2004). 15006352 10.1016/s0092-8674(04)00205-3

[b25] Zhang Z. & Dietrich F. S. Mapping of transcription start sites in *Saccharomyces cerevisiae* using 5′ SAGE. Nucleic Acids Res. 33, 2838 (2005). 15905473 10.1093/nar/gki583 PMC1131933

[b26] Struhl K. Promoters, activator proteins, and the mechanism of transcription initiation in yeast. Cell 49, 295 (1987). 2882858 10.1016/0092-8674(87)90277-7

[b27] Hahn S. & Young E. T. Transcriptional regulation in *Saccharomyces cerevisiae*: transcription factor regulation and function, mechanisms of initiation, and roles of activators and coactivators. Genetics 189, 705 (2011). 22084422 10.1534/genetics.111.127019 PMC3213380

[b28] Hahn S. Structure and mechanism of the RNA polymerase II transcription machinery. Nat. Struct. Mol. Biol. 11, 394 (2004). 15114340 10.1038/nsmb763 PMC1189732

[b29] Leuther K. K., Bushnell D. A. & Kornberg R. D. Two-dimensional crystallography of TFIIB– and IIE–RNA polymerase II complexes: implications for start site selection and initiation complex formation. Cell 85, 773 (1996). 8646784 10.1016/s0092-8674(00)81242-8

[b30] Carninci P. . Genome-wide analysis of mammalian promoter architecture and evolution. Nat. Genet. 38, 626 (2006). 16645617 10.1038/ng1789

[b31] Rosenkrantz M., Kell C. S., Pennell E. A., Webster M. & Devenish L. J. Distinct upstream activation regions for glucose-repressed and derepressed expression of the yeast citrate synthase gene Cit1. Curr. Genet. 25, 185 (1994). 7923403 10.1007/BF00357161

[b32] Curran K. A., Karim A. S., Gupta A. & Alper H. S. Use of expression-enhancing terminators in *Saccharomyces cerevisiae* to increase mRNA half-life and improve gene expression control for metabolic engineering applications. Metab. Eng. 19, 88 (2013). 23856240 10.1016/j.ymben.2013.07.001 PMC3769427

[b33] Van Slyke C. & Grayhack E. J. The essential transcription factor Reb1p interacts with the CLB2 UAS outside of the G2/M control region. Nucleic Acids Res. 31, 4597 (2003). 12888520 10.1093/nar/gkg638 PMC169905

[b34] Teixeira M. C. . The YEASTRACT database: an upgraded information system for the analysis of gene and genomic transcription regulation in *Saccharomyces cerevisiae*. Nucleic Acids Res. 42, D161 (2014). 24170807 10.1093/nar/gkt1015 PMC3965121

[b35] Stewart A. J., Hannenhalli S. & Plotkin J. B. Why transcription factor binding sites are ten nucleotides long. Genetics 192, 973 (2012). 22887818 10.1534/genetics.112.143370 PMC3522170

[b36] Harbison C. T. . Transcriptional regulatory code of a eukaryotic genome. Nature 431, 99 (2004). 15343339 10.1038/nature02800 PMC3006441

[b37] Erb I. & van Nimwegen E. Transcription factor binding site positioning in yeast: proximal promoter motifs characterize TATA-less promoters. PLoS ONE 6, e24279 (2011). 21931670 10.1371/journal.pone.0024279 PMC3170328

[b38] Mohibullah N. & Hahn S. Site-specific cross-linking of TBP *in vivo* and *in vitro* reveals a direct functional interaction with the SAGA subunit Spt3. Genes Dev. 22, 2994 (2008). 18981477 10.1101/gad.1724408 PMC2577793

[b39] Bhaumik S. R. & Green M. R. Differential requirement of SAGA components for recruitment of TATA-box-binding protein to promoters *in vivo*. Mol. Cell. Biol. 22, 7365 (2002). 12370284 10.1128/MCB.22.21.7365-7371.2002 PMC135674

[b40] Dudley A. M., Rougeulle C. & Winston F. The Spt components of SAGA facilitate TBP binding to a promoter at a post-activator-binding step in vivo. Genes Dev. 13, 2940 (1999). 10580001 10.1101/gad.13.22.2940 PMC317152

[b41] Xi L. . Predicting nucleosome positioning using a duration Hidden Markov Model. BMC Bioinformatics 11, 346 (2010). 20576140 10.1186/1471-2105-11-346 PMC2900280

[b42] Lee W. . A high-resolution atlas of nucleosome occupancy in yeast. Nat. Genet. 39, 1235 (2007). 17873876 10.1038/ng2117

[b43] Lee W. . A high-resolution atlas of nucleosome occupancy in yeast. Nat. Genet. 39, 1235 (2007). 17873876 10.1038/ng2117

[b44] Ganapathi M. . Extensive role of the general regulatory factors, Abf1 and Rap1, in determining genome-wide chromatin structure in budding yeast. Nucleic Acids Res. 39, 2032 (2011). 21081559 10.1093/nar/gkq1161 PMC3064788

[b45] Badis G. . A new library of yeast transcription factor motifs reveals a widespread function for Rsc3 in targeting nucleosome exclusion at promoters. Mol. Cell 32, 878 (2008). 19111667 10.1016/j.molcel.2008.11.020 PMC2743730

[b46] Leavitt J. & Alper H. Advances and current limitations in transcript-level control of gene expression. Curr. Opin. Biotechnol. 34, 98 (2015). 25559200 10.1016/j.copbio.2014.12.015

[b47] Curran K. . Short, synthetic terminators for improved heterologous gene expression in yeast. ACS Synth. Biol. doi:10.1021/sb5003357 (2015). 25686303

[b48] Sambrook J. & Russell D. W. Molecular cloning: a laboratory manual Cold Spring Harbor Laboratory Press (2001).

[b49] Gietz R. D. & Schiestl R. H. High-efficiency yeast transformation using the LiAc/SS carrier DNA/PEG method. Nat. Protoc. 2, 31 (2007). 17401334 10.1038/nprot.2007.13

[b50] Lee S. M., Jellison T. & Alper H. S. Systematic and evolutionary engineering of a xylose isomerase-based pathway in *Saccharomyces cerevisiae* for efficient conversion yields. Biotechnol. Biofuels 7, 122 (2014). 25170344 10.1186/s13068-014-0122-x PMC4147937

