# Supplementary material for: The development and characterization of synthetic minimal yeast promoters
Source: Nat Commun. 2015 Jul 17;6:7810. doi: 10.1038/ncomms8810 (PMC4518256; doi:10.1038/ncomms8810)
Supplement: Supplementary Information — Supplementary Figures 1-13 and Supplementary Tables 1-2 [file ncomms8810-s1.pdf]

Supplementary Figures

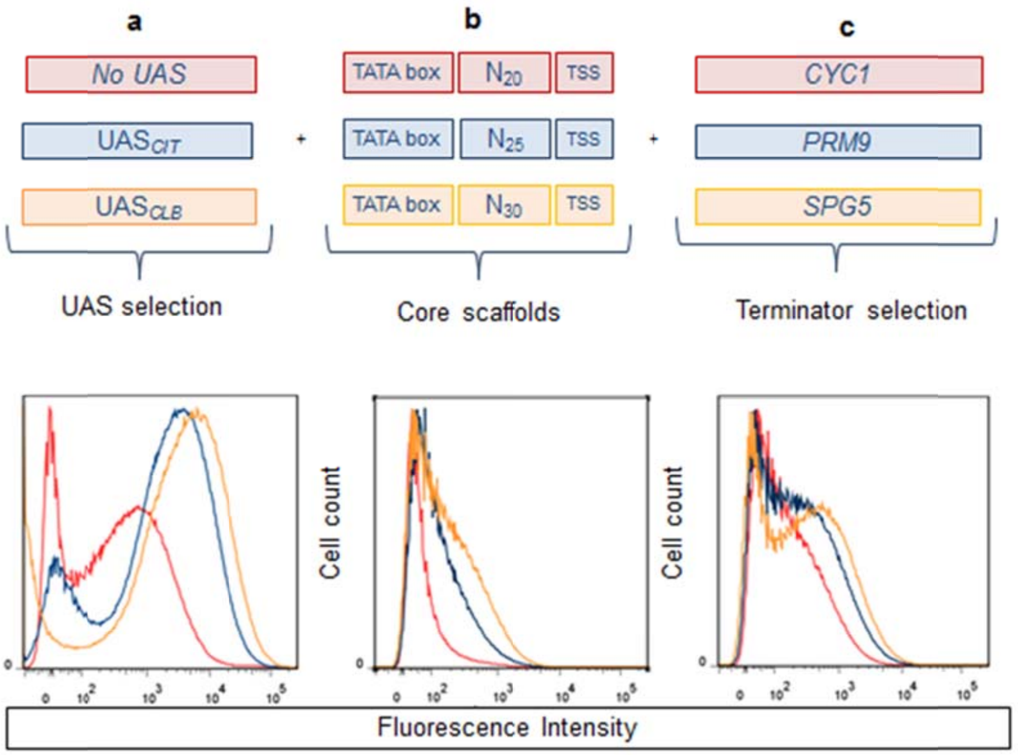

Supplementary Figure 1: *Promoter scaffold library assemblies*. Many ensembles of libraries were evaluated in this work. As a legend, the box outline color in top half of the figure corresponds to the line color used in the histograms in bottom half of figure. (**a**)Two UAS (UAS$_{CLB}$ and UAS$_{CIT}$) were used to distinguish functional candidates from non-functioning candidates. All libraries showed a similar shift, with UAS$_{CLB}$ eliciting the strongest shift towards higher fluorescence. Libraries shown are N$_{30}$-*SPG5*, UAS$_{CIT}$-N$_{30}$-*SPG5*, and UAS$_{CLB}$ -N$_{30}$-*SPG5* . (**b**)Three lengths of cores were tested, where oligonucleotides of 20 bp (N$_{20}$), 25 bp (N$_{25}$), and 30 bp (N$_{30}$) were placed between TATA box and TSS. Libraries shown here are N$_{20}$-*CYC1*, N$_{25}$-*CYC1*, and N$_{30}$-*CYC1* without a UAS. An increase in the spacing results in a tail lengthening of the histograms. (**c**)Expression enhancing terminators (those thought to increase mRNA half-life) were used to distinguish functional candidates from non-functioning candidates. *SPG5* produced a select population shift greater than other terminators tested. Shown here are libraries N$_{30}$-*CYC1*, N$_{30}$-*PRM9*, and N$_{30}$-*SPG5*.

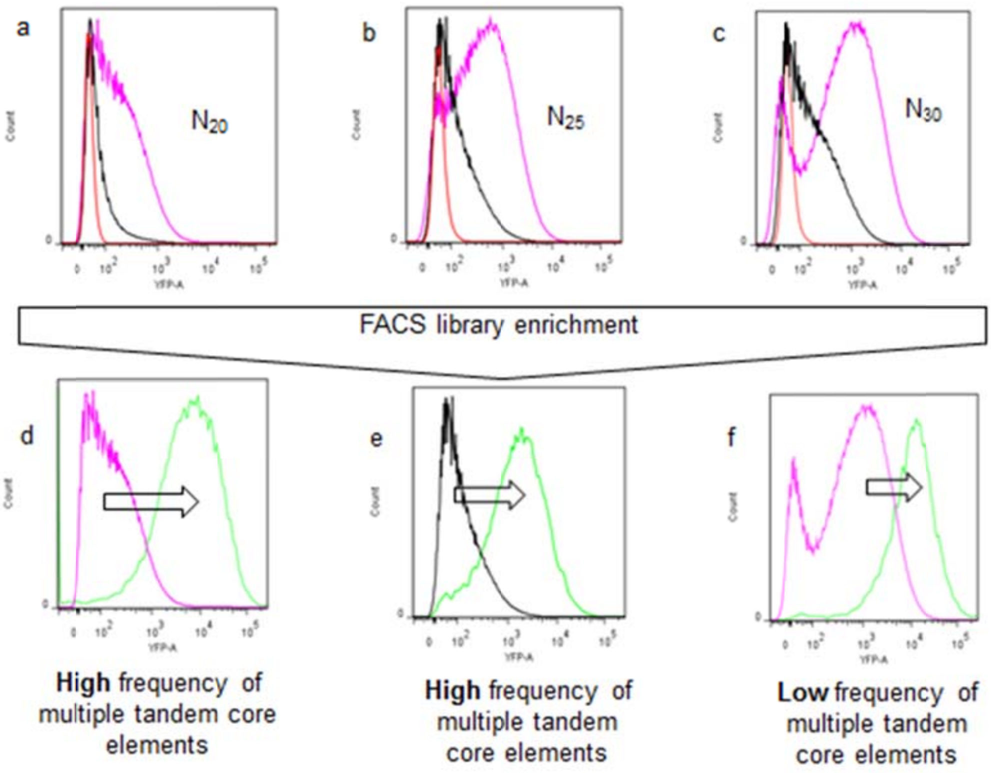

Supplementary figure 2: *Histograms of select libraries before and after sorting by FACS.* The top 0.15% fluorescent cells of each library was sorted by fluorescence activated cell sorting (FACS). (**a-c**) Flow cytometry histograms of unsorted libraries were gathered and overlaid. Red histograms represent unsorted negative controls (no yECitrine) (**a-c**), (**e**). Black histograms represent unsorted libraries without a UAS; N_{20}, N_{25} and N_{30} for (**a**), (**b, e**) and (**c**) respectively. Purple histograms represent UAS_{CIT} coupled with N_{20}, N_{25} and N_{30} for (**a, d**), (**b**) and (**c, f**) respectively. Histograms of sorted libraries were gathered and overlaid with unsorted respective libraries (**d-f**). Arrow in (**d-f**) indicates histogram shift due to FACS with histogram to left that of unsorted population. Unsorted populations shown in panels (**d-f**) color correspond to unsorted populations in panels (**a-c**) respectively. Only a few select sorted libraries are shown. Although histograms shifts are present after all library sortings (**d-f**) (compared to unsorted libraries), only libraries with N_{30} spacing produced libraries with a low frequency of tandem insertions.

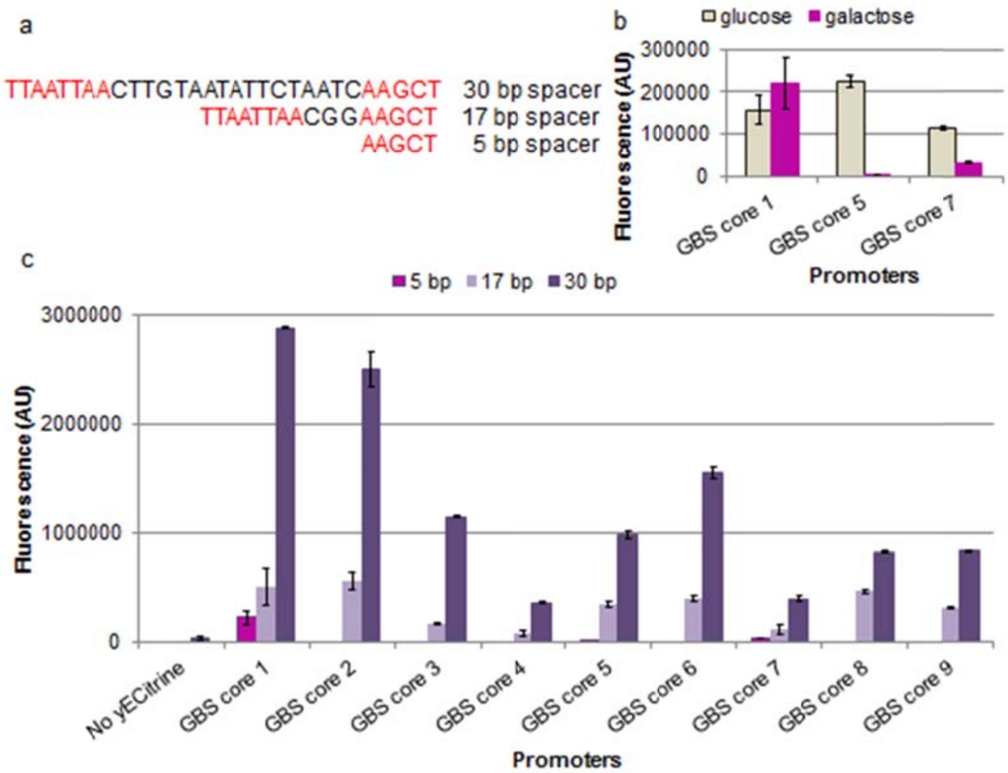

Supplementary Figure 3: *AT-rich spacer is required for galactose induction of core element by Gal4p binding site (GBS).* (**a**) Sequences of spacers used to distance GBS from core element. Restriction enzyme sites are indicated by red font. (**b**)No induction was observed when GBS was spaced just 5 bp upstream of the core element (n=3). In fact, two of the three promoters tested showed a reduction in expression under galactose induction. (**c**)Thus, GBS was positioned upstream of all core elements 17 bp and 30 bp, with the latter distance yielding the largest induction. Shown here is data for UAS_{G4BS4} coupled with core elements under galactose induction (n=3). Error bars represent standard deviation among biological triplicate.

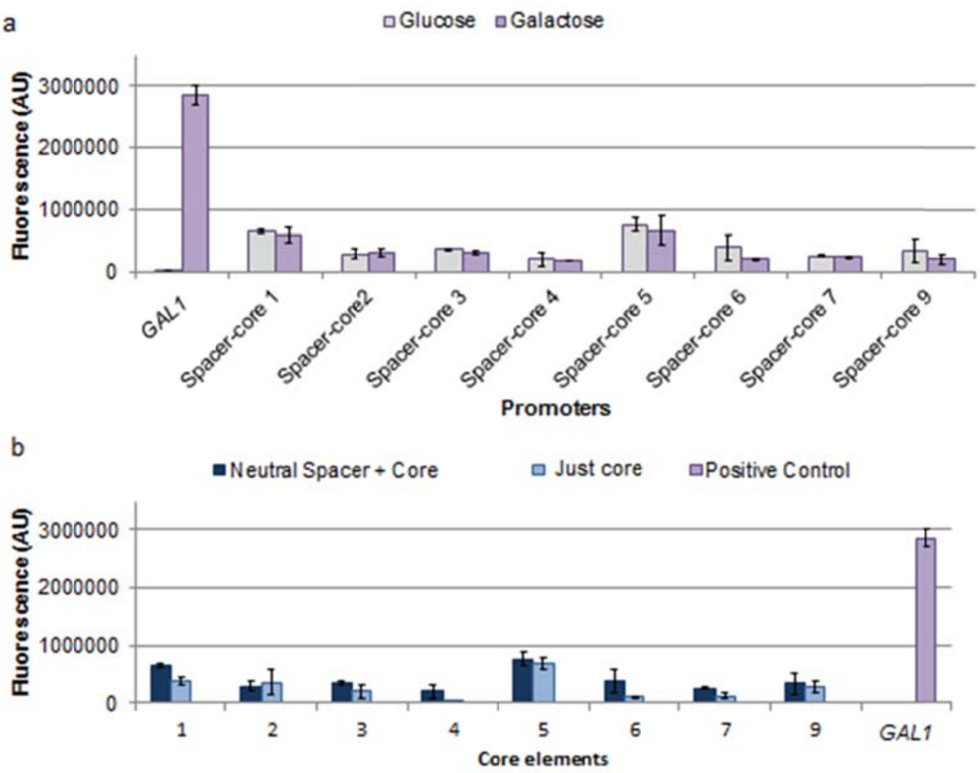

Supplementary Figure 4: *AT-rich spacer is neutral under glucose and galactose media for all core elements tested.* AT-rich spacer was designed to be free of TFBS as determined by YEASTRACT, and free of TATA boxes and TATA-like boxes (up to 2 mismatches to TATAWAWR). (**a**)30 bp neutral spacer does not respond to galactose induction (n=3). (**b**)30 bp neutral spacer has little to no effect on the core element's expression under glucose (n=3). Error bars represent standard deviation among biological triplicate.

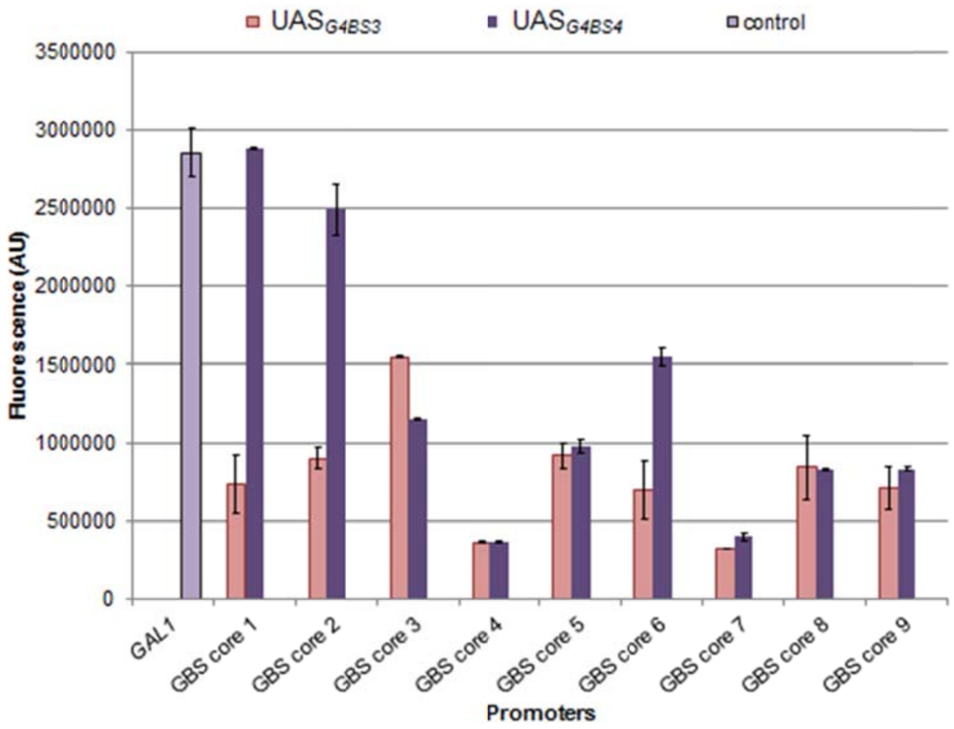

Supplementary Figure 5: *UAS_{G4BS4} shows increased induction over UAS_{G4BS3} under galactose induction*. Induction by various minimal GBS demonstrates both differential UAS function as well as generic function of the core elements (n=3). *GAL1* promoter serves as the positive control. Error bars represent standard deviation among biological triplicate.

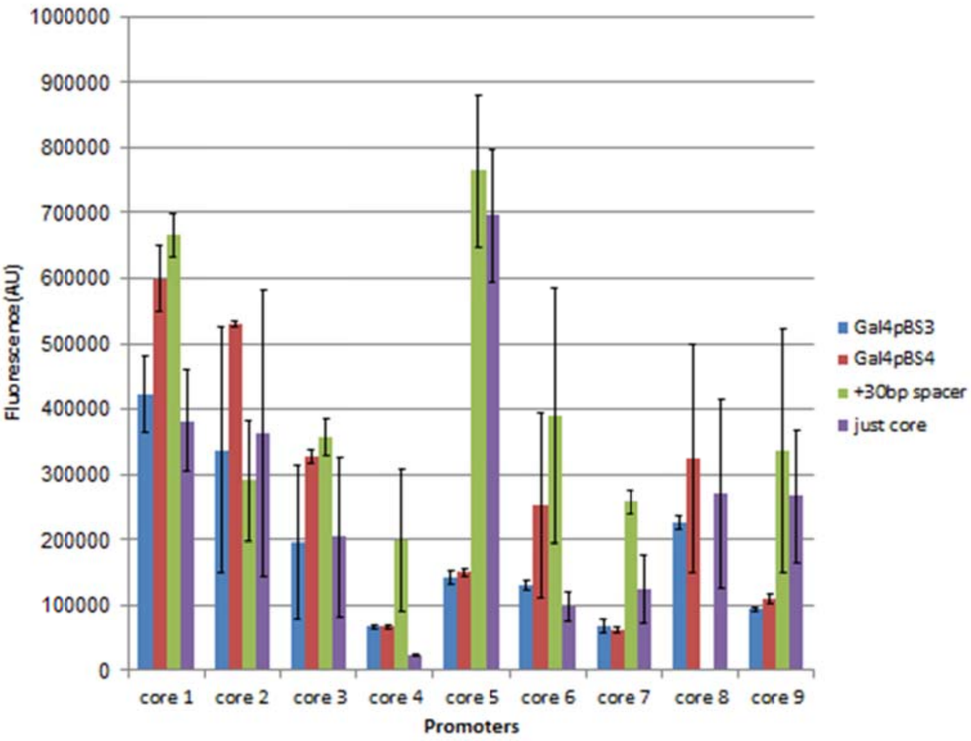

Supplementary Figure 6: *UAS_{G4BS3} and UAS_{G4BS4} have little to no effect on expression of core elements where glucose is sole carbon source.* Error bars represent standard deviation among biological triplicate (n=3).

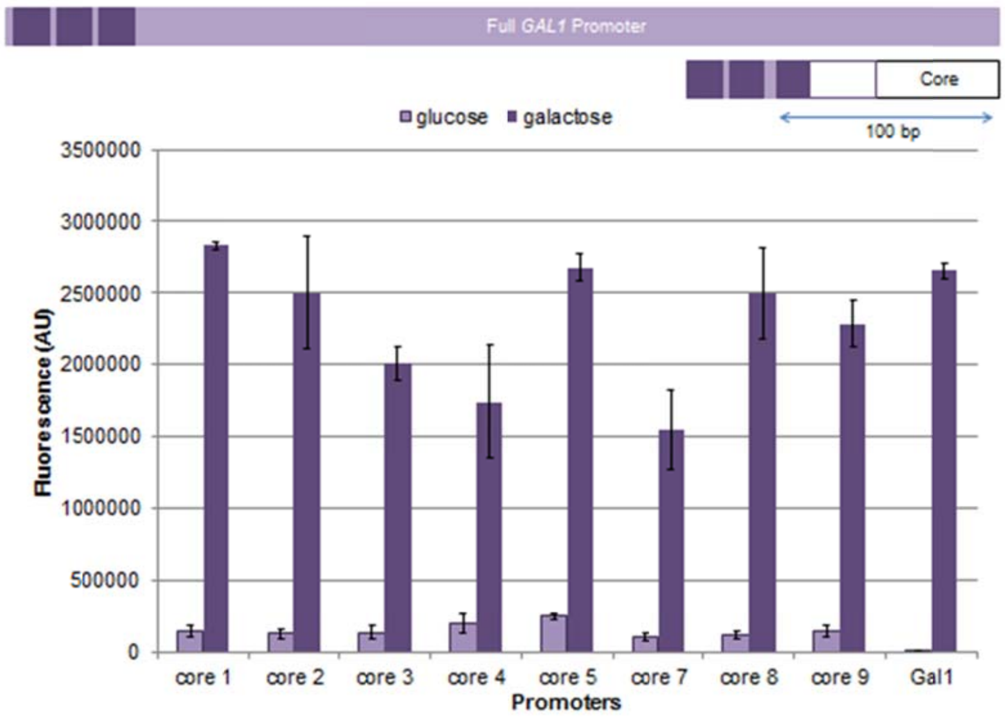

Supplementary Figure 7: *Multiple Gal4p binding sites provide strong inducibility.* A 54 bp sequence stretch derived from the 5' end of the native *GAL1* promoter containing three Gal4p binding sites (dark purple boxes) linked with core elements create strong galactose inducible promoters (n=3). Promoters depicted as boxes are drawn to scale with respect to length._Error bars represent standard deviation among biological triplicate.

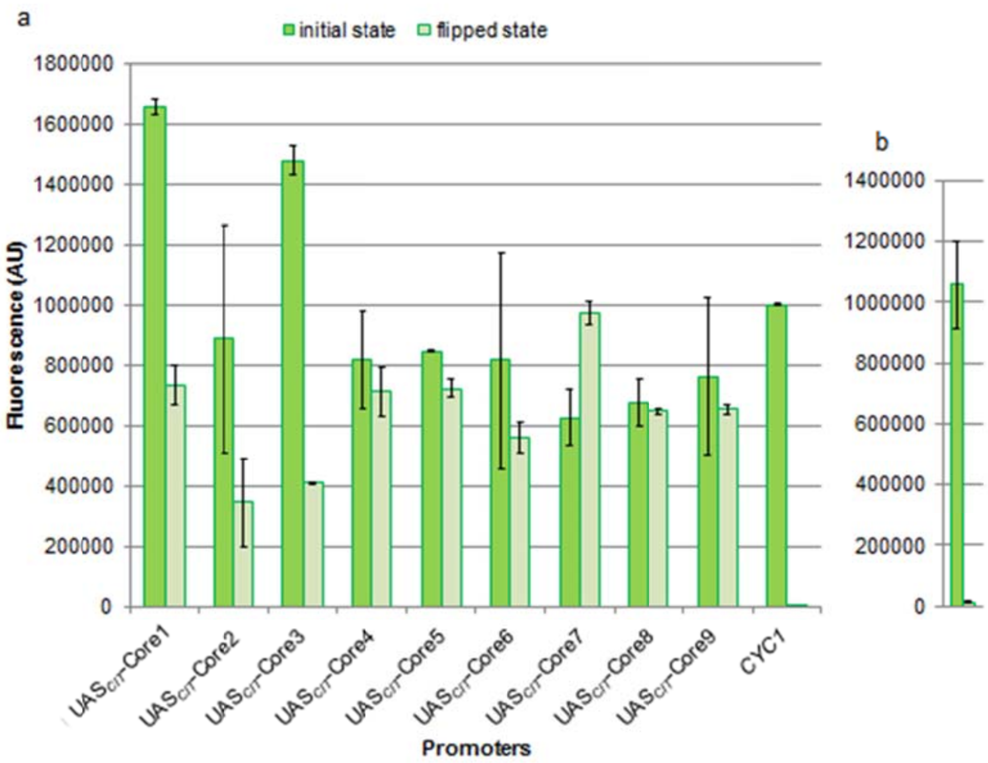

Supplementary Figure 8: *Promoters made using the core elements show less context specificity than commonly used CYC1 promoter*. (**a**) *CYC1* promoter's function is completely abolished when positioned differently within plasmid, whereas most promoters showed little to no effect (n=3). Of the 18 core elements subjected to this test, four were determined to be context dependent. For demonstrative purposes, one of these rejected core elements is shown (**b**) (n=3). Error bars represent standard deviation among biological triplicate.

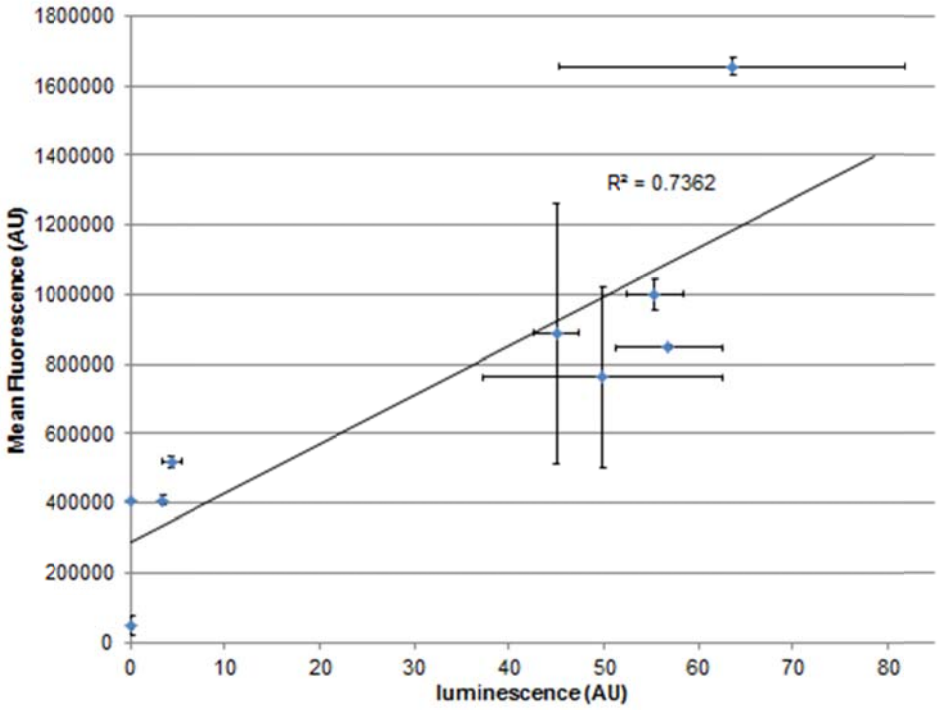

Supplementary Figure 9: *Mean fluorescence from flow cytometry parallels luminescence from LacZ assay under glucose growth.* This data supports the premise that these promoter elements are independent of the gene being expressed (n=3). Error bars represent standard deviation among biological triplicate.

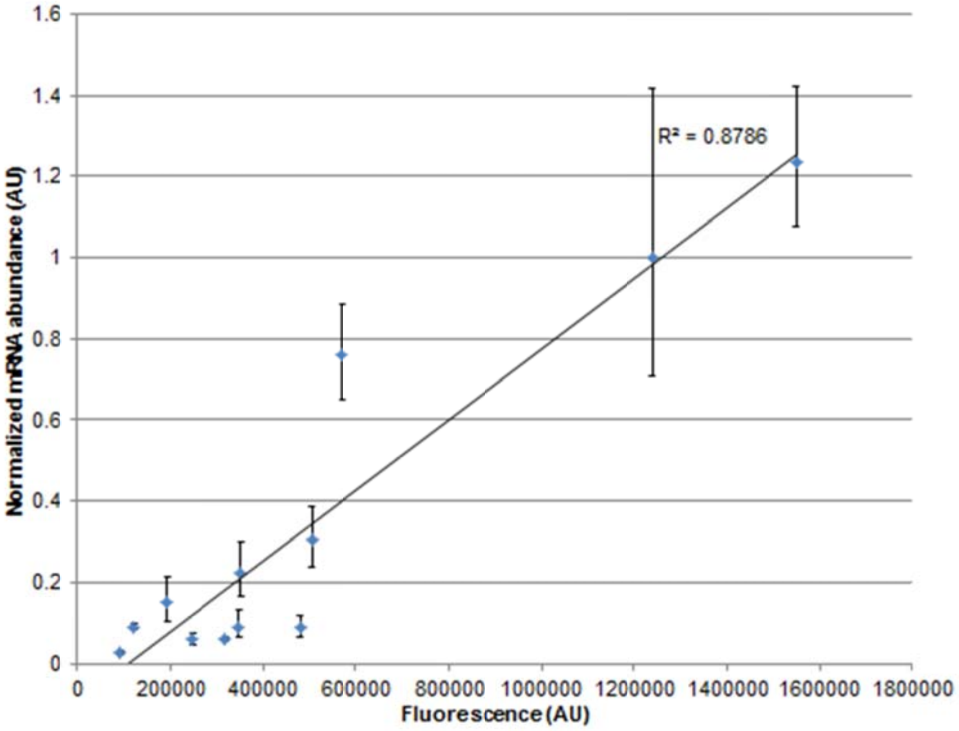

Supplementary Figure 10: *Fluorescence driven by synthetic promoters correlate with mRNA abundance*. mRNA levels supports the premise that these elements are functioning at the transcriptional level (n=3). Error bars represent standard deviation among biological triplicate.

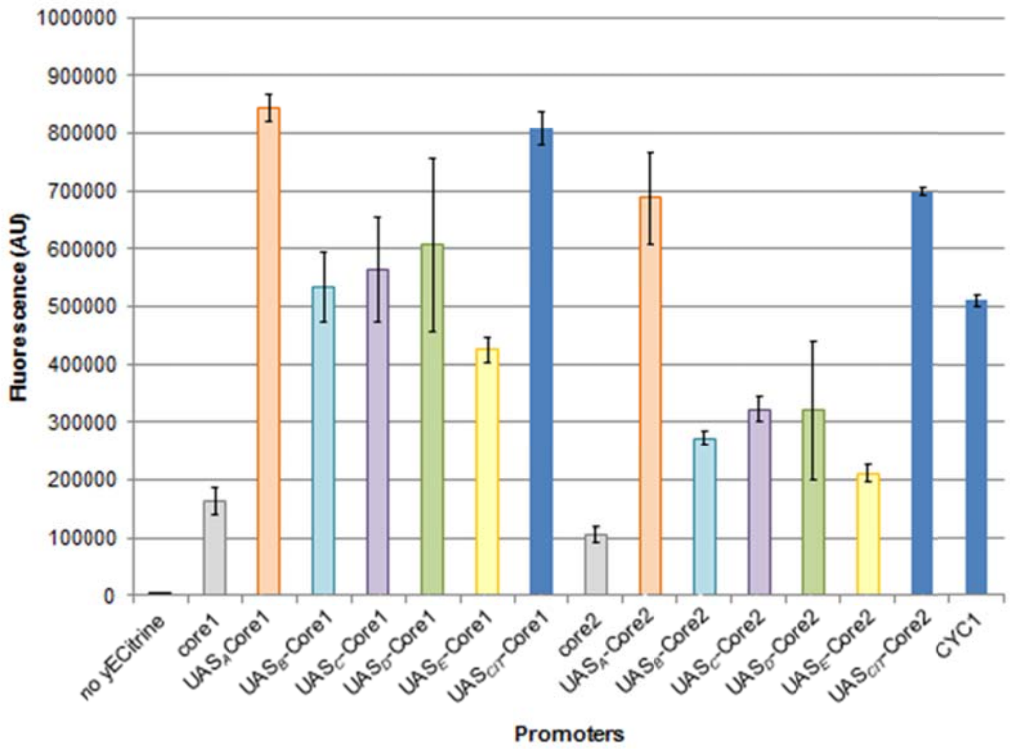

Supplementary Figure 11: *Synthetic UAS are generic*. 10 bp UAS elements derived from core 1 library function with core 2 (n=3). Error bars represent standard deviation among biological triplicate.

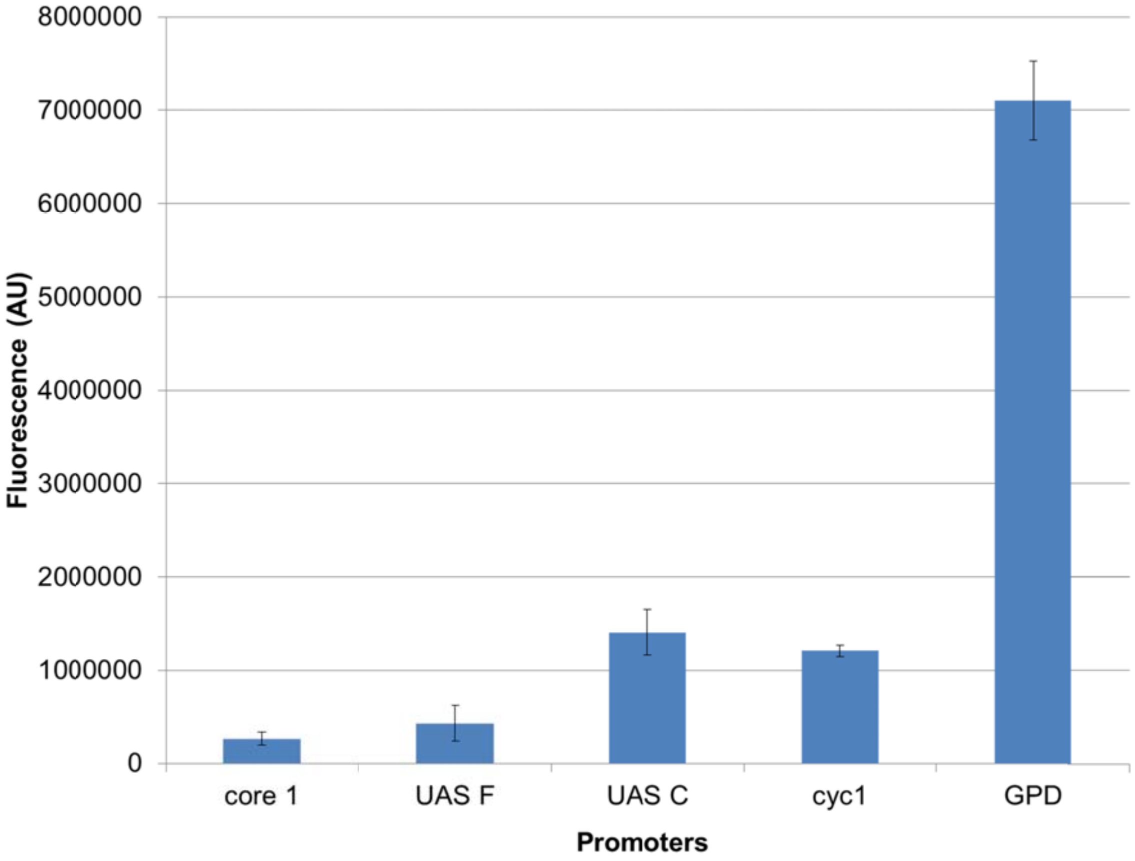

Supplementary Figure 12: *Fully synthetic minimal promoters function in the genomic context.* Several constructs were tested in a genomic context. Synthetic promoters had expression traits similar to those found in the promoter constructs. Error bars represent standard deviation among biological triplicate (n=3).

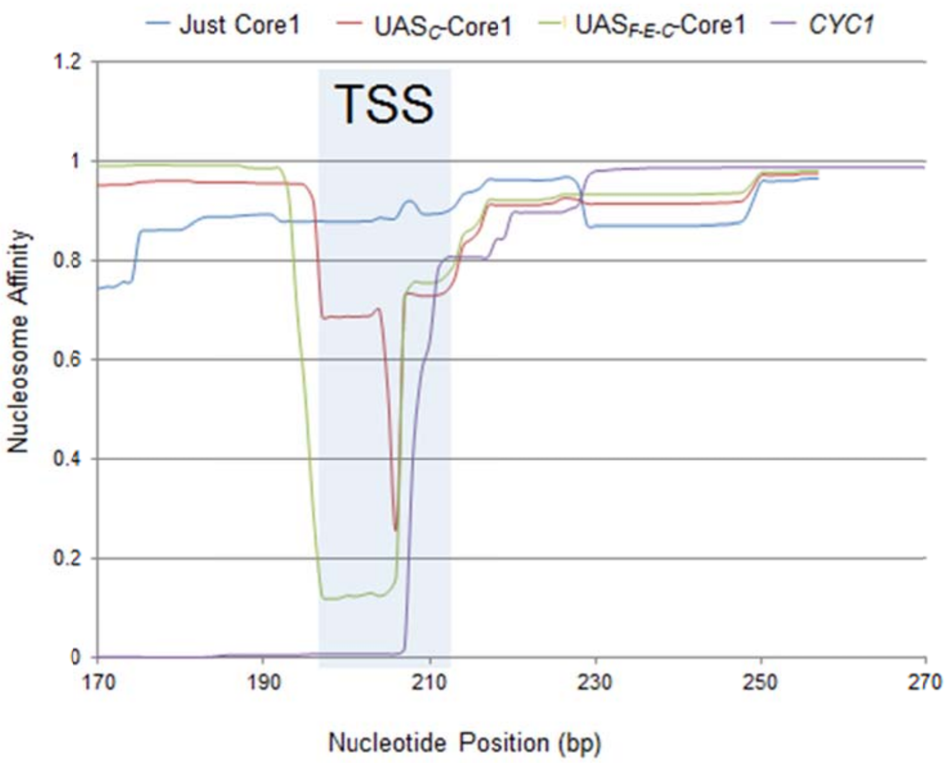

Supplementary Figure 13: *Synthetic UAS induce a predicted nucleosome depleted region at TSS*. Predictions of nucleosome occupancy via a hidden Markov Model demonstrate that UAS elements may be creating a nucleosome free region in the TSS area.

Supplementary Tables

Supplementary Table 1: *Oligonucleotides used in library assemblies, cloning and qPCR*

oligo	Description	Sequence
1	Fwrd primer- PCR *VSP13* term	acgcGGATCCTCACATATGAAAGTATATACCCGCTTTTGT
2	Rv primer- PCR *VSP13* term	cagGGCGCGCCCGCGCTGCGGATATTTCTAA
3	Fwrd primer- PCR *SPG5* term	gcgtcctcgagCAAAGACGTTGTTTCATCGC
4	Rv primer- PCR *SPG5* term	GAC GCG GTA CCG CTT ATT TTC TGC CGA ATT TTC AT
5	Fwrd primer- PCR *PRM9* term	gcgtcctcgagACAGAAGACGGGAGACACT
6	Rv primer- PCR *PRM9* term	ATT TTC AAC ATC GTA TTT TCC GAA GC G GTA CCG ACG C
7	Fwrd primer- PCR *CYC1* term	GCGTCaagctttcatgtaattagttatgtcacgcttaca
8	Rv primer- PCR *CYC1* term	GAC GCT CTA GAC GAG CGT CCC AAA ACC TT
9	20nt library oligonucleotide	GCGTCaagctTATAAAAGNNNNNNNNNNNNNNNNNNNNAAAAAAGCATCGAA AAAATCTAGActag
10	30nt library oligonucleotide	GCGTCaagctTATAAAAGNNNNNNNNNNNNNNNNNNNNNNNNNNNNNNAAAA AAGCATCGAAAAAATCTAGActag
11	25nt library oligonucleotide	GCGTCaagctTATAAAAGNNNNNNNNNNNNNNNNNNNNNNNNNAAAAAAGCA TCGAAAAAATCTAGActag
12	Primer to double strand libraries	CTA GTC TAG ATT TTT TCG ATG CTT TTT T
13	Fwrd primer- PCR expression cassettes for flip	gcgtcctcgagCAAAGACGTTGTTTCATCGC
14	Rv primer- PCR expression cassettes for flip	GAC GCG GTA CCG CTT ATT TTC TGC CGA ATT TTC AT
15	UAS library oligo	GGCGCGCCNNNNNNNNNNTTAATTAActtgtaatattctacccAAGCTTggg
16	Primer used to double strand oligo15	CCC AAG CTT GGG TAG AAT ATT ACA AGT TAA TTA A
17	Fwrd primer- PCR *CYC1* promoter	gcgtcAAGCTTatttggcgagcgttgg

18	Rv primer- PCR *CYC1* promoter	GAC GCT CTA GAT TAG TGT GTG TAT TTG TGT TTG C
19	Fwrd primer- PCR *GPD* promoter	GAC GCT CTA GAA TCC GTC GAA ACT AAG TT
20	Rv primer- PCR *GPD* promoter	GCGTCaagcttagtttatcattatcaatactcgccattt
21	Fwrd primer- PCR *TEF1* promoter	atcattGGCGCGCCatagcttcaaaatgtttctactcctttttttactcttc
22	Rv primer- PCR *TEF1* promoter	aaggtctagaaaacttagattagattgctatgctttctttctaatgagc
23	Fwrd primer- PCR *GAL1* promoter	gcgtcGGCGCGCCtagtacggattagaagccgccg
24	Rv primer-PCR *GAL1* promoter	GAC GCT TAA TTA AGT TTT TTC TCC TTG ACG TTA AAG TAT AGA GGT
25	Oligo of G4BS4 for 5 bp spacing	gcgtcttaattaaCGGAAGACTCTCCTCCGaagcttgcgtc
26	Oligo to double strand oligo 25	GAC GCA AGC TTC GGA GGA GAG TCT TCC GTT AAT TAA GAC
27	Oligo of G4BS3 for 5 bp spacing	gcgtcttaattaaCGGGCGACAGCCCTCCGaagcttgcgtc
28	Oligo to double strand oligo 27	GAC GCA AGC TTC GGA GGG CTG TCG CCC GTT AAT TAA GAC GC
29	Oligo of G4BS4 for 17 bp spacing	gcgtcGGCGCGCCCGGAAGACTCTCCTCCGTTAATTAAgcgtc
30	Oligo to double strand oligo 29	GAC GCT TAA TTA ACG GAG GAG AGT CTT CCG GGC GCG CCG ACG C
31	Oligo of G4BS3 for 17 bp spacing	gcgtcGGCGCGCCCGGGCGACAGCCCTCCGTTAATTAAgcgtc
32	Oligo to double strand oligo 31	GAC GCT TAA TTA ACG GAG GGC TGT CGC CCG GGC GCG CCG ACG C
33	Oligo of AT-rich neutral spacing	gcgtcTTAATTAActtgtaatattctaatcAAGCTTgcgtc
34	Oligo to double strand oligo 33	GAC GCA AGC TTG ATT AGA ATA TTA CAA GTT AAT TAA GAC GC
35	Oligo of $UAS_{F\text{-}E\text{-}C}$	atcattGGCGCGCCCCTCCTTGAAACTGAAATTTTAGCATGTGATTAATTAAggccg
36	Primer to double strand oligo 35	CGG CCT TAA TTA ATC ACA TGC TAC ACC GCC CCC
37	Fwrd primer- qPCR of yECitrine	TTCTGTCTCCGGTGAAGGTGAA
38	Rv primer- qPCR of yECitrine	TAAGGTTGGCCATGGAACTGGCAA
39	Fwrd primer- qPCR of Alg9	ATCGTGAAATTGCAGGCAGCTTGG
40	Rv primer- qPCR of Alg9	CATGGCAACGGCAGAAGGCAATAA

Supplementary table 2: *Libraries assembled*. A catalogue of the libraries assembled in this work.

UAS	TATA-TSS spacing (bp)	Terminator	*E. Coli* library size (10^6)	Yeast cells isolated by FACS
None	20	*CYC1*	0.3	1350
None	30	*CYC1*	0.2	574
UAS_{CIT}	20	*CYC1*	2.2	3700
UAS_{CIT}	30	*CYC1*	1.4	456
UAS_{CLB}	20	*CYC1*	3.0*	Not sorted
UAS_{CLB}	30	*CYC1*	2.4*	Not sorted
None	20	*SPG5*	0.7	9874
None	30	*SPG5*	0.7	1463
UAS_{CIT}	20	*SPG5*	0.9	1300
UAS_{CIT}	30	*SPG5*	0.1	285
UAS_{CLB}	20	*SPG5*	0.1*	Not sorted
UAS_{CLB}	30	*SPG5*	0.04*	Not sorted
None	20	*PRM9*	0.5*	Not sorted
None	30	*PRM9*	0.7	31
UAS_{CIT}	20	*PRM9*	0.04	640
UAS_{CIT}	30	*PRM9*	0.1	325
UAS_{CLB}	20	*PRM9*	2.0*	Not sorted
UAS_{CLB}	30	*PRM9*	0.6*	Not sorted
None	25	*SPG5*	1.0	23323
None	25	*PRM9*	1.0	1469
None	25	*CYC1*	0.9	1418
UAS_{CIT}	25	*SPG5*	2.4	4250
UAS_{CIT}	25	*PRM9*	0.9	3232
UAS_{CIT}	25	*CYC1*	2.3	2900
N_{10}-spacer	30	*SPG5*	1.3	140

*This library size was not factored into the 15 million elements created since it was not sorted and analyzed.
